# Quantitative Electroencephalographic Biomarkers for Repetitive Transcranial Magnetic Stimulation Treatment Response Prediction in Mild Cognitive Impairment: A Pilot Study Protocol for Multi‐Center, Assessor‐Blinded, Open‐Label Clinical Trial

**DOI:** 10.1002/brb3.71017

**Published:** 2025-10-31

**Authors:** Zahra Yousefian, Seyed Hamzeh Hosseini, Mohammad Ali Nazari, Reza Kazemi, Mohammad Asghari‐Jafarabadi, Hamed Ghazvini, Raheleh Rafaiee, Seyedeh Masoumeh Seyedhosseini Tamijani

**Affiliations:** ^1^ Student Research Committee, School of Advanced Technologies in Medicine Mazandaran University of Medical Sciences Sari Iran; ^2^ Department of Neuroscience, School of Advanced Technologies in Medicine Mazandaran University of Medical Sciences Sari Iran; ^3^ Psychosomatic Research Center Mazandaran University of Medical Sciences Sari Iran; ^4^ Department of Neuroscience, Faculty of Advanced Technologies in Medicine Iran University of Medical Sciences Tehran Iran; ^5^ Faculty of Entrepreneurship University of Tehran Tehran Iran; ^6^ Cabrini Research, Cabrini Health Malvern VIC Australia; ^7^ School of Public Health and Preventive Medicine Monash University Melbourne VIC Australia; ^8^ Department of Psychiatry, School of Clinical Sciences Monash University Clayton VIC Australia

## Abstract

**Rationale:**

Over 55 million people worldwide suffer from dementia, with 10 million new cases diagnosed annually. Due to the limited efficacy of drug therapies, alternative approaches like repetitive transcranial magnetic stimulation (rTMS) have gained popularity as a non‐invasive, safe method leveraging neural plasticity and brain connectivity. However, its high cost and time commitment highlight the need for biomarkers to predict treatment response.

**Aims:**

This pilot study aims to identify a quantitative electroencephalography (QEEG) biomarker to predict which mild cognitive impairment patients will respond to rTMS. By targeting responders early, clinicians can make rTMS more cost‐effective and time‐efficient, reducing wasted treatment on non‐responders.

**Design:**

This multi‐center, assessor‐blinded clinical trial will examine QEEG biomarkers as predictors of rTMS treatment responsiveness in 25 patients with mild cognitive impairment (MCI). Adults aged 60 years or older will undergo cognitive assessments using the Montreal Cognitive Assessment (MoCA) or mini‐mental state examination (MMSE) and have an electroencephalography (EEG) recording. Participants will complete 10 rTMS sessions targeting the left DLPFC over 2 weeks, with 2000 pulses per session at 20 Hz. Cognitive tests will be repeated post‐treatment, and participants will be classified as responders or non‐responders based on cognitive changes, then baseline QEEG parameters will be compared between the two groups. The primary endpoint is the proportion of responders at ten sessions after rTMS (score post‐intervention > score pre‐intervention = responder, according to the minimal clinically important difference (MCID) threshold (i.e., an increase of at least 3 points or 10% on the MMSE, or an increase of at least 1 point on the MoCA); score post‐intervention ≤ score pre‐intervention = non‐responder). The secondary endpoints are the differences in baseline QEEG features between responders and non‐responders.

**Outcome:**

By identifying responders prior to treatment, we can optimize resource allocation, minimize the time and cost associated with ineffective treatments, and ultimately improve the quality of care for individuals with MCI.

**Trial Registration:**

IRCT registration number: IRCT20240218061042N1 (version updated September 7, 2024)

## Graphical abstract

Repetitive transcranial magnetic stimulation (rTMS) enhances neural plasticity in mild cognitive impairment (MCI), but treatment success varies across individuals. In this pilot multicenter trial, quantitative EEG (QEEG) signals recorded before therapy are analyzed to identify predictors of clinical response. Patients receive 10 sessions of high‐frequency rTMS over the left DLPFC, and are classified as responders or non‐responders based on cognitive improvement. Baseline QEEG features distinguishing responders can serve as non‐invasive biomarkers to optimize rTMS resource allocation and personalize dementia care.

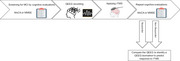

**Abbreviations**
MCI: Mild cognitive impairment
MMSE: Mini‐mental state examination
MoCA: Montreal Cognitive Assessment
QEEG: Quantitative electroencephalography
rTMS: Repetitive transcranial magnetic stimulation

Dementia is a pathological condition in which the decline in cognitive functions is greater than that of biological aging. Over 55 million people worldwide suffer from dementia, and the World Health Organization (WHO) estimates that 10 million new cases will be diagnosed annually (Bai et al. 2022; Organization). By 2050, this population is predicted to rise to 131.5 million (Prince et al. 2016). The WHO report from 2019 estimates that dementia costs the global economy 1.3 trillion (Organization 2025).

Cholinergic and glutamatergic medications have poor efficacy as therapies for any type of dementia, indicating the necessity for novel therapeutic and preventive strategies. Mild cognitive impairment (MCI) is a transitional, preclinical, asymptomatic state before dementia (Zhang et al. 2019). According to most studies conducted in this field, treatment interventions for mild cognitive impairment yield better and more successful treatment outcomes than those for the more severe forms of dementia (Rogers et al. 2023; Koch et al. 2020).

By leveraging neural plasticity mechanisms, including long‐term potentiation (LTP) and long‐term depression (LTD), transcranial non‐invasive brain stimulation has demonstrated efficacy in producing temporary changes in cognition, emotion, and behavior among individuals with mild to moderate cognitive impairment (Dayan et al. 2013; Moretti and Rodger 2022; Thomson and Sack 2020).

Transcranial magnetic stimulation (TMS) is a non‐invasive brain stimulation technique, a safe method with high tolerability and no major adverse effects such as diabetes, tardive dyskinesia, weight gain, etc. (Lefaucheur et al. 2017; Lefaucheur et al. 2020; Su et al. 2023). Furthermore, cortical excitability, neuroplasticity, and functional connectivity between different brain regions are also altered by repetitive TMS (rTMS) (Wassermann and Zimmermann 2012; Salomons et al. 2014). In TMS, action potentials in neurons can be physically induced by an electromagnetic field or a “pulse” produced by running an electric current through a coil (Barker et al. 1987; Barker et al. 1985). While rTMS has shown promise in improving cognitive function in some individuals with MCI, its efficacy is variable (Yan et al. 2023). Identifying reliable predictors of treatment response would be crucial to optimize treatment selection and improve patient outcomes (Lithgow et al. 2022).

In line with this goal, a 2024 comprehensive meta‐analysis of major depressive disorder research identified frontal theta activity in EEG as the most promising candidate for predicting treatment outcomes (Jin et al. 2024). Building on this finding, ongoing research continues to explore the potential of predictive biomarkers.

Proposed indices of usefulness of magnetic stimulation in major depressive disorder include large spectral power values within the low‐frequency θ2 (6–8 Hz) and α1 (8–9 Hz) sub‐bands in the posterior (parietal‐occipital‐posterior) EEG leads (Iznak et al. 2020), as well as the utilization of artificial neural networks with a sensitivity of 93.33% (Erguzel et al. 2015). Furthermore, it is well‐established that in Alzheimer's disease, the primary component of EEG abnormalities involves alterations in alpha and theta bands (Güntekin et al. 2020). The findings of the 2022 investigation are consistent with this observation, demonstrating that EEG event‐related theta responses may be susceptible to neuromodulation therapies (Kayasandik et al. 2022).

Electroencephalography appears to be the preferred paraclinical tool for identifying potential biomarkers of response to magnetic stimulation treatment due to its non‐invasive nature, high temporal resolution, and relative affordability. Furthermore, quantitative EEG (QEEG) further enhances the analysis of EEG data by providing objective and quantitative measures of brain activity, such as power spectral analysis and brain mapping (Fingelkurts and Fingelkurts 2022). This study is conceived as an exploratory investigation aimed at identifying preliminary QEEG biomarkers associated with treatment response to rTMS in individuals with MCI. Designed as a pilot, this study aims to provide foundational insights into the feasibility and potential direction of QEEG analysis in this context, estimating preliminary effect sizes and characterize signal patterns and identifying potential trends, and generating data to inform the design and sample size calculations of future, more definitive trials. By identifying likely responders prior to treatment, the study aspires to optimize resource allocation, reduce the time and costs associated with ineffective interventions, and ultimately improve the quality of care for individuals with MCI.

## 1 Study Design

**FIGURE 1:**
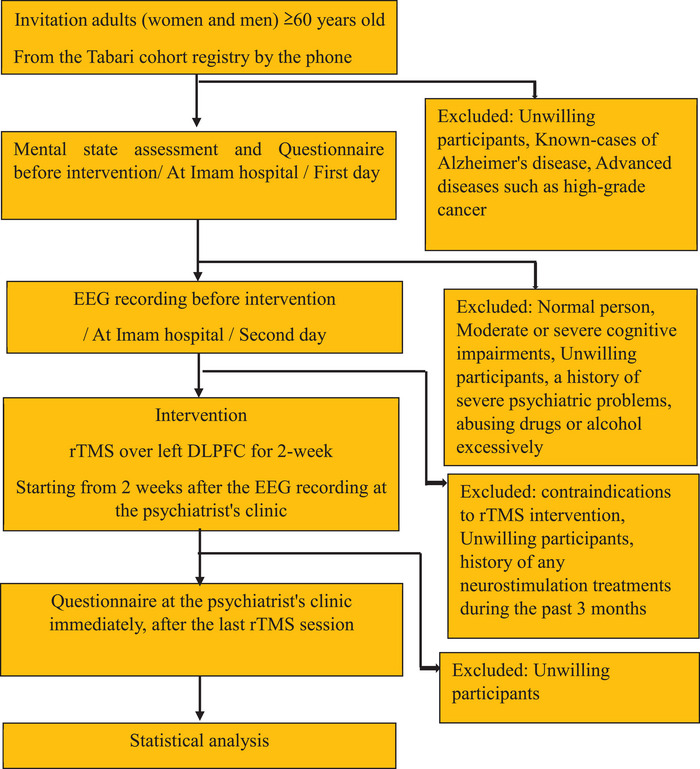
Study design.

This pilot, multicenter, assessor‐blinded study recruited adults aged ≥60 years from the Tabari cohort registry. After cognitive screening at Imam Hospital, eligible participants underwent baseline EEG recording. Two weeks later, they received 10 sessions of high‐frequency rTMS (20 Hz) over the left DLPFC at the psychiatrist's clinic. Post‐intervention cognitive testing was performed to classify responders and non‐responders (Fig. 1). The full procedural sequence of the study procedure is described in detail below.

This study design as shown in (Figure 1).

### 1.1 Participants

#### 1.1.1 Setting; Recruitment Method

PERSIAN Organizational Cohort Study (POCS) (Poustchi et al. 2018) is the collaborative participation of medical universities on a population of 200,000 Iranians from all races and ethnicities. The Tabari cohort is a subset of the POCS. The Tabari cohort consists of 4199 adults who voluntarily enrolled in the study. Participants undergo annual follow‐ups conducted by specialists and faculty members from various clinical and basic science departments at Mazandaran University of Medical Sciences. This registry project is conducted free of charge and aims to identify diseases and risk factors. All demographic information of volunteers, including address, telephone number, and medical history, is in this registry (Kheradmand et al. 2019). All participants in the Tabari cohort will be contacted by telephone. After providing a detailed description of the study protocol, adults aged 60 years and older (both women and men) who are registered in the Tabari cohort will be invited to undergo global cognitive screening, provided they are able to attend the visit at Imam Hospital, taking into account any physical or mobility limitations. After completing a demographic checklist (e.g., age range, gender, education level) on the first day of the visit, all individuals without any exclusion criteria undergo a mental state assessment and cognitive tests in line with the cohort group's facilities. However, individuals identified with MCI who are currently eligible to participate in the study according to the study's inclusion and exclusion criteria will be thoroughly informed about the study and legal rights and sign informed consent forms. The next day, after EEG recording, patients who consent to rTMS intervention will then complete a screening questionnaire, supervised by a psychiatrist, prior to the treatment. The participants will all be eligible for TMS operation (Rossi et al. 2009; Mokhtarinejad et al. 2024) based on their responses to a TMS safety screening questionnaire and will then complete an Iranian version of the 28‐item General Health Questionnaire (GHQ) (Ebrahimi et al. 2007).

#### 1.1.2 Eligibility Criteria

Inclusion criteria: (1) Persian language. (2) have the ability to visit the hospital. (3) all patients aged 60 years or older. (4) participants will be diagnosed with MCI in the Diagnostic and Statistical Manual of Mental Disorders‐5th edition (DSM‐V) and will be additionally assessed by the Montreal Cognitive Assessment (MoCA) (Badrkhahan et al. 2020) and the Mini‐Mental State Examination (MMSE) (Seyedian et al. 2008; Delbari et al. 2024) “22<MoCA<26 or 20<MMSE<24.” (5) not using of psychiatric or neurological medications. (6) patients who will sign informed consent forms.

Exclusion criteria: (1) a history of severe psychiatric problems such as a major depressive disorder or psychosis. (2) history of any neurostimulation treatments during the past 3 months. (3) contraindications to rTMS intervention, such as the presence of a cardiac pacemaker and ferromagnetic implants in the head or neck. (4) history of serious neurological illnesses, including epilepsy, seizures, or stroke. (5) abusing drugs or alcohol excessively. (6) with a skull fracture or craniotomy.

Patients diagnosed with MCI will be invited for EEG recording the next day. After EEG recording, those who consent to rTMS intervention will complete a screening questionnaire, supervised by a psychiatrist, prior to the treatment. The participants will all be eligible for TMS operation (Rossi et al. 2009; Mokhtarinejad et al. 2024) based on their responses to a TMS safety screening questionnaire. The TMS Safety Screening Questionnaire will be used to evaluate the eligibility for TMS intervention based on the questionnaire from Rossi et al. (2021), covering questions about the existence of risk factors, such as epilepsy or the presence of a cardiac pacemaker and ferromagnetic implants in the head or neck. According to their responses, patients will be categorized as either “Eligible,” or “Not eligible.”

Then complete an Iranian version of the 28‐item General Health Questionnaire (GHQ) (Ebrahimi et al. 2007). The General Health Questionnaire (GHQ‐28) is a general health screening tool, and obtaining a score of 24 (Ebrahimi et al. 2007) or higher is a condition for volunteers to continue participating in the study, as well as not having any TMS contraindications.

#### 1.1.3 Informed Consent Process

Informed consent will be received by the investigator involved in this study. Patients will be completely informed in comprehensible terms of the requirements, the risks, the objectives, and safety measures. They will be informed of their right to withdraw at any time without incurring any penalty and to refuse to participate. All of this information is explained in the consent form that will be provided to the participant. The investigator will collect written consent of the participant prior to final inclusion in the study.

### 1.2 Assessment Tools

#### 1.2.1 Questionnaire

The most popular test for diagnosing dementia is the MMSE, whereas the Montreal Cognitive Assessment (MoCA) is a very accurate test for mild cognitive impairment. To assess volunteers' global cognition, we will use MMSE and MoCA.

Thirty questions make up the MMSE, which evaluates orientation (10 points), immediate recall (3 points), attention and calculation (5 points), recall (3 points), and language (9 points) (Seyedian et al. 2008; Abe et al. 2020).

Eight cognitive domains are evaluated by the thirty questions in the MoCA, which also include executive functions and visuospatial skills (5 points), attention (6 points), naming (3 points), abstract thinking (2 points) language (3 points), recall (5 points), and orientation (6 points) (Badrkhahan et al. 2020). Higher scores on the MMSE and MoCA indicate higher cognitive function; scores range from 0 to 30. Because of the nature of questionnaires, if participants are uneducated (0 year of schooling), we add 3 points to the score of MMSE. Range of the Farsi version of thr MMSE is 20 < MMSE < 24, indicating MCI (Delbari et al. 2024). Besides, in MOCA test, Because the nature of questionnaires, if the subjects have less than 12 years of schooling (1‐11 years of schooling), we add 1 point to the score and range of 22 < MoCA < 26 indicate MCI (Zhao et al. 2011). For educated individuals, we will use MOCA exams. and for uneducated subjects (0 years of schooling), we will only use the MMSE test.

Finally, a history and mental state assessment by a psychiatrist based on the Diagnostic and Statistical Manual of Mental Disorders, fifth edition (DSM‐V), is crucial because the decline or stability of cognitive performance compared to the past is the most important issue (Edition 2013; Kaplan 2015).

MMSE and MoCA tests will be repeated at the end of the treatment to classify participants as responders or non‐responders to the intervention. We will use the minimal clinically important difference (MCID), based on clinically meaningful changes in cognitive test scores. The MCID denotes the smallest change in score perceived as beneficial or significant by patients or clinicians. Detailed thresholds for the MMSE and MoCA tests are comprehensively described in the post‐intervention assessment section.

#### 1.2.2 EEG Recording

QEEG is software that quantitatively extracts EEG data for numerical analysis. QEEG parameters: The scalp records the electrical potentials associated with the brain, known as EEG. The four rhythms of delta, theta, alpha, and beta are considered the main frequency bands of brain waves. Delta rhythm covers the frequency range from 1 to 4.0 Hz, theta (4–8 Hz), alpha (8–12 Hz), broad beta (12–25 Hz), and hi‐beta (25–30 Hz). All frequency rhythms take into account the following parameters: absolute power, relative power, power ratio, peak frequency, amplitude asymmetry, coherence, and phase lag.

✓ Absolute power: The amount of wave energy in a specific frequency spectrum in the frequency domain.
✓ Relative power: Divide the absolute power of a specific frequency band by the total absolute power of all frequency bands.
✓ Power ratio: Divide the power of one frequency band by the power of another frequency band.
✓ Peak frequency: The peak detection method for computerized EEG analysis involves measuring the amplitude and time interval between successive maxima (peaks) and minima (crossings) in a signal.
✓ Amplitude asymmetry: There are neuroanatomical differences between the left and right sides of the brain, as well as lateral functional differences, also known as lateralization of brain function. Asymmetry is usually calculated by subtracting the natural logarithm of the left hemisphere‐specific frequency power from the right hemisphere‐specific frequency power natural logarithm while using link ears as the reference electrode.

There are neuroanatomical differences between the left and right sides of the brain, as well as lateral functional differences, also known as lateralization of brain function. Asymmetry is usually calculated by subtracting the natural logarithm of the left hemisphere‐specific frequency power from the right hemisphere‐specific frequency power natural logarithm while using linked ears as the reference electrode.

✓ Coherence: Coherence represents the correlation coefficient between two signals, which can be a measure of the phase stability between two signals at a certain frequency and can take a value between 0 and 1. A coherence of zero indicates very large phase changes between two signals, while a Coherence of 1 indicates that the two signals have a constant phase difference over time.
✓ Phase lag: A method for estimating connectivity in EEG in a way that excludes volume conduction effects. The phase difference for a sinusoidal waveform is the amount of angular displacement of the waveform relative to a given reference point on the horizontal axis. This angle is measured in degrees or radians. In other words, the phase difference between two waves is the difference between two waveforms relative to a common axis.

To obtain these parameters, first, the assembly of all the recorded data is defined based on the linked‐ear assembly. Then, Neuro‐Guide software is used to convert brain waves into quantitative data.

In this study, all EEG recordings will be done between 2:00 p.m. and 4:00 p.m. Persons with mild cognitive impairment will be invited for an EEG recording the day after the cognitive examination. The day before the EEG recording, the subjects wash their heads in a bath to remove all residues from the scalp. We advise the instructed person not to change their usual daily routine before the EEG recording and to continue taking their main medication. After arriving at the EEG recording site, we give the subject time to rest and adapt to the environment. To avoid increased impedance due to sweating and soiling, the scalp will be cleaned once more using an alcohol pad, and all devices such as cell phones will preferably be kept away from the room because they produce disturbing noises. The room will be kept quiet certainly during EEG recording, and the EEG recording for each participant takes 20–30 min.

We will record the participants' EEG data using a 19‐channel EEG system with passive electrodes (Science Beam; Parto Danesh Co., Iran). It has 19 scalp channels, a sampling rate of 500 Hz per channel, an ADC resolution of 24 bits, and a CMRR of 75 dB at 500 Hz. The electrode placement will follow the 10–20 international norm and referential montage linked ears. Every subject had a 5‐minute resting‐state EEG recording with their eyes open, followed by a 5‐min recording with their eyes closed, all while maintaining an impedance of less than 10 kΩ and applying a 0.5 low‐cut and a 500‐Hz high‐cut filter.

Finally, after the automated algorithm, manual checking by an expert neuroscientist for the artifact rejection process. Topographic maps will be generated for each QEEG feature in the frequency domain (e.g., absolute power, relative power, power ratio, peak frequency, amplitude asymmetry, coherence, and phase lag) for both responder and non‐responder groups using Neuro Guide software version 3.2.1.1 (Loo et al. 2016; Coburn et al. 2006).

### 1.3 Intervention

#### 1.3.1 rTMS

TMS is a non‐invasive method that uses electromagnetic induction to stimulate the brain. By utilizing an insulated coil, TMS can depolarize or hyperpolarize neurons depending on the frequency of the applied stimulation. The magnetic pulses generated by the coil painlessly penetrate the scalp, bones, and brain membranes to reach the neurons, resulting in short‐term activity within the corresponding areas. Rapid and sequential delivery of these pulses, known as repetitive TMS, can induce longer‐lasting changes in brain activity (Sandrini et al. 2011). Furthermore, the pulse penetrates approximately 2.5 cm below the coil, affecting neurons in distant regions through synapses (Sharbafshaaer et al. 2024; Paganin and Contini 2025). In this treatment method, several key parameters are established, including the following: the stimulation site in the brain, the duration of stimulation, the interval between sessions, the total number of sessions, the number of pulses in each session, the pulse frequency, and the intensity (Chou et al. 2020; Zhang et al. 2021; Šimko et al. 2022). We will use the Magstim Rapid2‐Biphasic Stimulator (Magstim, Whitland, UK) equipped with the Double 70 mm Air Film Coil (AFC). We will first explain to participants the common and rare, but serious, side effects of rTMS and ask them to sign a consent form. Common side effects may include headaches (30%), facial muscle twitching (30%), lightheadedness or dizziness after treatment (20%), discomfort or pain at the stimulation site (20%), and fatigue (1%–7%). These side effects are usually mild, typically resolve during treatment, and can be alleviated by resting or taking over‐the‐counter pain medication such as ibuprofen or acetaminophen. Another possible side effect that occurs very rarely is an epileptic seizure (less than 0.1%) (Taylor et al. 2021). We will record all side effects reported by participants during the study. All procedures will be conducted between 4:00 p.m. and 8:00 p.m., as hormone levels related to the circadian cycle, such as melatonin and cortisol, can influence brain plasticity. Research indicates that the effectiveness of repetitive transcranial magnetic stimulation (rTMS) is higher when administered in the afternoon compared to the morning (Abo and Kakuda 2015). In the initial phase, it is imperative to ascertain the resting motor threshold (RMT), which will be evaluated based on the minimal intensity of stimulation applied to the primary motor cortex necessary to elicit a reliable motor‐evoked potential (MEP) in the first dorsal interosseous (FDI) muscle over five successive single pulses. It is important to set this threshold for each participant individually (Rossini et al. 2015) using established methods (Schutter and van Honk 2006). During each treatment session, the patient sits in a specially designed chair, similar to a dental unit. Before starting the treatment, the patient must remove all objects that are sensitive to magnetism, such as credit cards and jewelry around the head and neck, because a magnetic field will be created. Additionally, if possible, the patient will be given hearing protectors (in‐ear pads) to prevent any potential harm from the sound of the pulses.

The rTMS intervention will be initiated 2 weeks after the EEG recording. On the first day, we will mark the stimulation site, specifically the left DLPFC, on the scalp using the 5.5 cm method. Another approach for localizing the left DLPFC is called the Beam F3 method. Many studies have declared that the effectiveness and reliability of these two methods in targeting the DLPFC are comparable (Pace et al. 2023; Trapp et al. 2023). We will conduct a total of 10 treatment sessions, with 1 session per day for 5 consecutive days over 2 weeks. Each session will involve delivering 2000 pulses at a frequency of 20 Hz, with a cycle of 2 s on and 28 s off, lasting a total of 25 min. We will target 100% RMT. These parameters were selected based on a recent review suggesting that they can produce the optimal clinical outcome (Lefaucheur et al. 2014). Trained PhD students in neuroscience will continuously monitor rTMS sessions, including pulse rate, respiratory rate, and any adverse events.

We target the DLPFC because it is a key region for higher‐level cognition, and its pathological damage leads to a characteristic feature of MCI (Yan et al. 2023; Xie et al. 2021). Moreover, it plays a crucial role in some major brain networks, such as the central executive network (CEN) and the fronto‐parietal network (FPN), indicating that the left DLPFC is the most effective site to improve global cognition outcomes (Opitz et al. 2016; Agosta et al. 2012).

**FIGURE 2:**
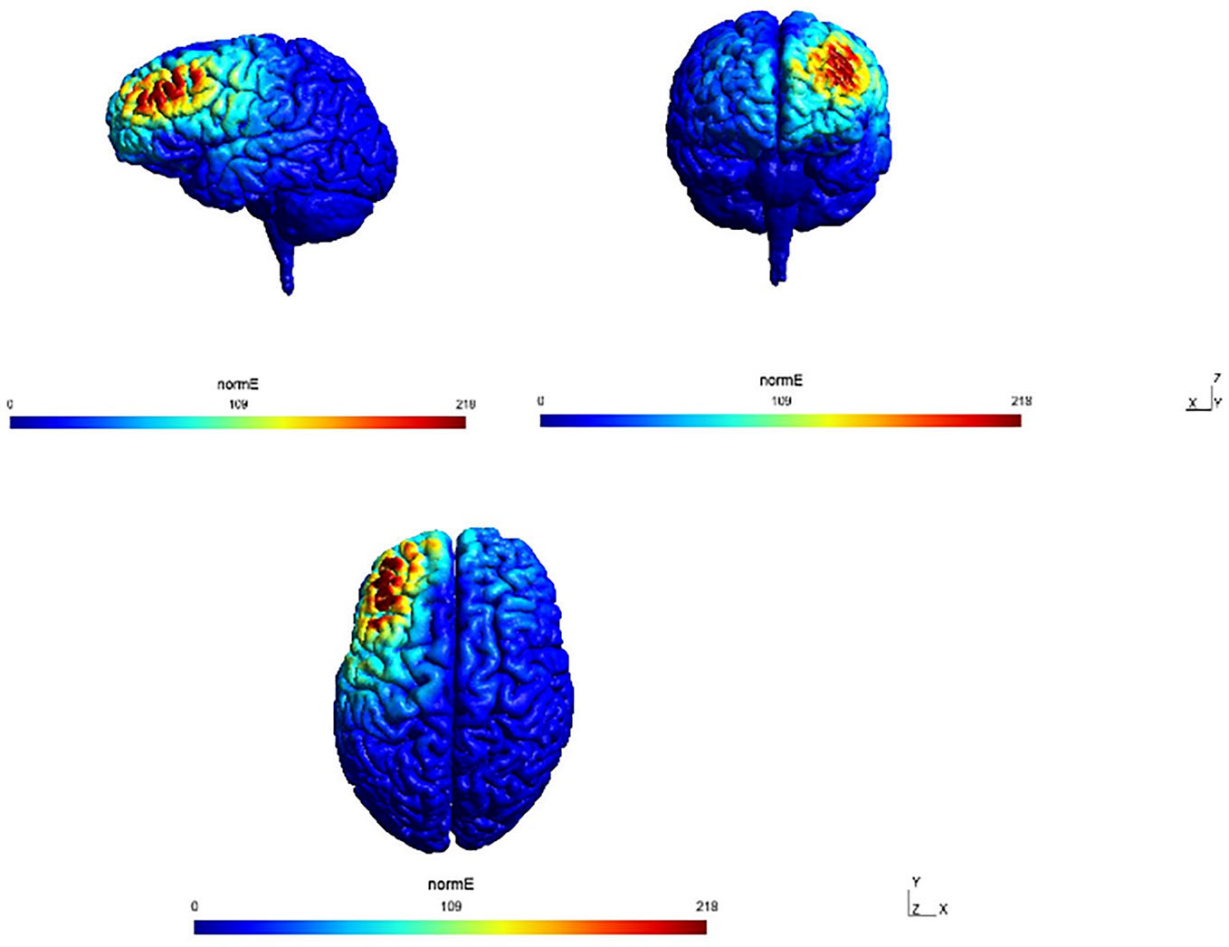
Simulation depicting the normalized magnetic field applied by rTMS following our protocol, simulated using SimNIBS, highlighting the maximal induced field over the left DLPFC.

The SimNIBS‐3.2 toolbox was applied to investigate the efficacy of the electric field produced by rTMS through the left DLPFC under our protocol (Figure 2) (Thielscher et al. 2015).

### 1.4 Post Intervention Assessment

#### 1.4.1 Questionnaires

After 10 rTMS sessions, we will perform the cognitive tests again under the same conditions. For educated individuals, we will use MoCA exams, and for uneducated subjects (0 years of schooling), we will only use the MMSE test again. If a participant's MMSE or MoCA score increases after the TMS, they will be considered a responder. Conversely, if their score decreases or there are no changes, they will be classified as a non‐responder (Kayasandik et al. 2022).

We will utilize the minimal clinically important difference (MCID) to evaluate significant changes in test scores. According to the MCID, an increase of 3 points or 10% (Borland et al. 2022; Watt et al. 2021) in the MMSE indicates cognitive improvement (Borland et al. 2022; Watt et al. 2021). The MCID for the MoCA may vary, but we will consider a 1‐point increase as a sign of cognitive improvement (Lindvall et al. 2024). After completing the work, patients will receive free visits from the psychosomatic fellowship to continue their treatment in accordance with ethical principles.

### 1.5 Group Classification

MCI with 22 < MoCA < 26 or 20 < MMSE < 24 after 2 weeks of intervention and post‐assessments will be divided into two groups (score post‐intervention > score pre intervention = responder, according to MCID threshold (i.e., an increase of at least 3 points or 10% on the MMSE, or an increase of at least 1 point on the MoCA); score post‐intervention ≤ score pre‐intervention = non‐responder).

### 1.6 Endpoint Measures

#### 1.6.1 Primary Endpoint

The primary endpoint is the proportion of responders according to MCID. Additionally, the primary analysis will determine which cognitive subdomains show the greatest therapeutic effects.

#### 1.6.2 Secondary Endpoint

The secondary outcome will be differences in baseline QEEG features between the responder and non‐responder groups. By identifying responders prior to treatment, we can optimize resource allocation, minimize the time and cost associated with ineffective treatments, and ultimately improve the quality of care for individuals with MCI.

### 1.7 Statistical Methods

#### 1.7.1 Primary Endpoint

All assessors will be blinded to group allocation and receive data labeled as “group A” and “group B.” Statistical analyses will be conducted using StataNow18.5 (Stata Corp, College Station, TX, USA), adhering to CONSORT guidelines. Normality of continuous variables will be checked using skewness and kurtosis, with variables considered non‐normal if absolute skewness > 2 or kurtosis > 3. Baseline characteristics, including (sex, hyper‐lipidemia, hypothyroid, hyper‐tension, diabetes, smoking, ischemic heart disease, education, and age) will be compared between groups using Fisher's exact test for categorical variables and the Mann–Whitney *U* test for non‐normal continuous variables. For normally distributed continuous variables, between‐group comparisons will be performed using the independent samples t‐test.

Initially, paired *t*‐tests (a suitable transformation or non‐parametric test) or equivalents for non‐normal data will be used to evaluate overall pre‐ to post‐rTMS changes within all 25 participants, assessing global treatment efficacy and identifying cognitive subdomains demonstrating the greatest response. Subsequently, participants will be categorized into responders and non‐responders based on MCID criteria. Between‐group comparisons of cognitive scores and subdomains will then be performed using ANOVA for baseline and ANCOVA for post‐intervention outcomes, adjusting for baseline scores and potential confounders. Pre‐intervention values refer specifically to baseline cognitive test scores, totally and in each cognitive sub‐domain, and related continuous variables measured before rTMS, which serve as covariates in the analyses. The QEEG features are analyzed separately as part of the secondary endpoint assessment. For non‐normal variables, suitable transformation will be applied, and median differences with percentage changes reported. Marginal plots from ANCOVA will visualize adjusted group differences post‐intervention. This two‐stage analytical approach allows comprehensive evaluation of rTMS effects both globally and within clinically meaningful responder groups across cognitive domains.

#### 1.7.2 Secondary Endpoint

Topographic maps will be generated for each QEEG feature (e.g., absolute power, relative power, power ratio, peak frequency, amplitude asymmetry, coherence, and phase lag) for both responder and non‐responder groups using Neuro Guide software, then calculate the average QEEG features for each group using Neuro Guide. Subsequently, the statistical significance of the dissimilarity between the two groups' EEG topographic maps will be determined using independent *t*‐tests or the Mann–Whitney *U* test, depending on the normality of the variables. ROC analysis estimating Area Under the Curve (AUC), sensitivity (SN), specificity (SP), positive predictive value (PPV), negative predictive value (NPV), A positive likelihood ratio (LR+), and the negative likelihood ratio (LR‐) for optimal cut‐points, as well as comparing AUC across biomarkers, will be performed using Stata 18 software. These diagnostic accuracy measures specifically assess the predictive ability of QEEG biomarkers in distinguishing responder from non‐responder status.

## 2 Discussion

In this study, QEEG baseline in MCI patients will be used for the first time to discriminate responders that obtained cognitive improvements after rTMS from non‐responders.

The global prevalence of dementia is predicted to triple in the next three decades (Prince et al. 2016), and currently there appears to be no disease‐modifying therapy or cure for dementia on the immediate horizon (Middleton et al. 2018).

Several studies suggest that rTMS can improve general cognitive function (Kayasandik et al. 2022; Šimko et al. 2022; Koch et al. 2022). However, it is important to note that TMS is a time‐consuming and expensive procedure that requires patients to make daily visits over an extended period of 4 to 6 weeks (Goodman et al. 2024). These factors represent a significant challenge for patients requiring this treatment. Use of the device is ineffective in some individuals, resulting in wasted time for effective treatment of individuals who do not respond. Identifying a QEEG biomarker that can predict recovery prior to intervention would be highly valuable. As the number of FDA approvals for this device increases and interest grows, the importance of predictive biomarkers will also rise. Investigating changes in QEEG parameters related to dementia presents a fascinating area of research within neuroscience.

Research has indicated that in cases of mild dementia, there is an increase in the theta brainwave band coupled with a decrease in the beta brainwave band. As dementia progresses, this pattern is further characterized by an increase in the delta brainwave band and a decrease in the alpha brainwave band (Gandelman‐Marton et al. 2017). The increase in the delta brainwave band corresponds to the white matter shrinking in the frontal region of patients with MCI and Alzheimer's disease (Babiloni et al. 2006). Additionally, the reduction of the alpha brainwave band occurs in the parietal, occipital, and temporal regions, in line with hippocampal shrinkage in MCI and Alzheimer's patients (Babiloni et al. 2009). Furthermore, the use of acetylcholinesterase inhibitors in dementia and Alzheimer's treatment leads to a reduction in the delta and theta brainwave bands (Jelic et al. 1998). Progression of disease and the failure of pharmacological treatments are associated with an increase in the delta and theta brainwave bands and a decrease in the alpha and beta brainwave bands (Jelic et al. 1998). Patients with Alzheimer's disease also show an increase in low‐frequency brainwave band power and a decrease in high‐frequency brainwave band power (Baggio et al. 1991). Additionally, there is a significant decrease in coherence in the theta, alpha, and beta brainwave bands in the frontal regions, which is consistent with the loss of neurons and disconnection of neocortical neurons (Besthorn et al. 1994).

Previous research with similar objectives has shown that patients with reduced levels of consciousness exhibit a higher absolute power of alpha waves in both the left and right parietal regions, which is associated with a greater likelihood of responding to rTMS (He et al. 2020). Additionally, in patients with severe dementia, the difference in the theta band has been observed as a predictor for respondents (Kayasandik et al. 2022). However, these studies differ from ours in terms of the stimulation parameters, including the stimulation site and disorder. Our findings will contribute to the development of correct prescription of rTMS therapy for MCI patients.

Besides, there are some limitations to this protocol study. As an open‐label trial, it is subject to significant confounds, including treatment expectations on the part of participants, but this is a methodological limitation intrinsic to an open‐label trial (Thase 2001). For the future direction, more analysis with a larger dataset will be recommended, and randomized, sham‐controlled study can affect the generalizability and reliability of the outcomes. Additional predictive biomarkers such as genetic factors, neuroimaging, or biochemical biomarkers can be noticed in future study.

## 3 Conclusion

This study aims to identify a QEEG biomarker that can serve as a predictor of recovery in patients with MCI before the commencement of the rTMS intervention. The rising concerns regarding the prevalence of significant cognitive disorders, coupled with the deficiency of effective treatment options, have intensified the focus on innovative technologies such as rTMS. However, its time‐intensive and expensive nature often hinders the application of TMS. Consequently, the development of predictive biomarkers that can anticipate therapeutic responses before the intervention is of considerable importance. Our research investigates cognitive improvement following rTMS and examines whether specific QEEG features differ between participants who improved (responders) and those who did not (non‐responders).

## 4 Limitations

One key limitation of this study is the relatively small sample size, particularly in the context of post hoc stratification into responders and non‐responders. Given the exploratory nature of the study and the anticipated imbalance in group sizes, the statistical power to detect large between‐group differences is limited. As such, the findings should be interpreted with caution and viewed primarily as preliminary indications of potential QEEG biomarkers associated with treatment response. This limitation constrains the generalizability of the results and precludes definitive hypothesis testing. However, this study serves as a pilot investigation intended to generate effect size estimates and feasibility data that will inform the design and power calculations of future, adequately powered confirmatory studies.

## Author Contributions

S.M.S.T., M.A.N., S.H.H., and Z.Y. made significant contributions to the conception and design of the study. S.M.S.T. contributed to the finalization of study procedures, and Z.Y. will contribute to data collection. Z.Y. drafted the manuscript, and all other authors were involved in revising it critically for intellectual content. M.A.N. will be the assessor of QEEG data, and M.A.‐J. will be responsible for the statistical analysis. R.K. contributed to the finalization of rTMS protocol parameters, H.G. will contribute to visualization, and R.R. will handle writing, review, and editing. All authors gave final approval of the version to be published and agree to be accountable for all aspects of the work.

## Ethics Statement

Under the identification IR.MAZUMS.REC.1402.18234, the ethics committee of Mazandaran University of Medical Sciences has accepted our study, which is being carried out in compliance with the Declaration of Helsinki. Besides, this study has been registered at the Iranian Registry of Clinical Trials with IRCT registration number IRCT20240218061042N1 (version updated September 7, 2024).

## Consent

All participants will either provide written informed consent to participate in the study or provide assent to have a substitution decision‐maker provide informed consent on their behalf.

## Funding

This work is supported by a grant from the Medical University of Mazandaran (18234).

## Conflicts of Interest

The authors declare no conflicts of interest.

## Peer Review

The peer review history for this article is available at https://publons.com/publon/10.1002/brb3.71017

## Data Availability

The datasets generated and analyzed during the current study are available from the corresponding author on reasonable request.
